# Estimating comparable English healthcare costs for multiple diseases and unrelated future costs for use in health and public health economic modelling

**DOI:** 10.1371/journal.pone.0197257

**Published:** 2018-05-24

**Authors:** Adam D. M. Briggs, Peter Scarborough, Jane Wolstenholme

**Affiliations:** 1 Nuffield Department of Population Health, University of Oxford, Oxford, Oxfordshire, United Kingdom; 2 The Dartmouth Institute for Health Policy and Clinical Practice, Dartmouth College, Lebanon, New Hampshire, United States of America; University of Groningen, NETHERLANDS

## Abstract

**Objectives:**

Healthcare interventions, and particularly those in public health may affect multiple diseases and significantly prolong life. No consensus currently exists for how to estimate comparable healthcare costs across multiple diseases for use in health and public health cost-effectiveness models. We aim to describe a method for estimating comparable disease specific English healthcare costs as well as future healthcare costs from diseases unrelated to those modelled.

**Methods:**

We use routine national datasets including programme budgeting data and cost curves from NHS England to estimate annual per person costs for diseases included in the PRIMEtime model as well as age and sex specific costs due to unrelated diseases.

**Results:**

The 2013/14 annual cost to NHS England per prevalent case varied between £3,074 for pancreatic cancer and £314 for liver disease. Costs due to unrelated diseases increase with age except for a secondary peak at 30–34 years for women reflecting maternity resource use.

**Conclusions:**

The methodology described allows health and public health economic modellers to estimate comparable English healthcare costs for multiple diseases. This facilitates the direct comparison of different health and public health interventions enabling better decision making.

## Introduction

Disease specific healthcare costs for use in health and public health economic models can be calculated using one of two different approaches: adding together the costs of all components of a patient’s care, such as staff and equipment (a micro-level or bottom-up approach), or allocating an overall healthcare budget to specific diseases (a macro-level or top-down approach).

Guidelines exist for how to estimate costs for health economic cost-effectiveness models, however these do not provide specific advice on how to identify costs when evaluating the impact on multiple diseases simultaneously where the comparability of data sources for different diseases is important [1,2]. Without comparable data sources, there is the potential for significant variation in disease costs due to either the inclusion or exclusion of different cost components, following patients for different lengths of time, or locally driven variations in patient pathways [3–6]. This is particularly relevant to public health economic models where an intervention may affect many diseases. For example, if comparing the cost-effectiveness of a salt reduction strategy with a sugar reduction strategy to decide how to prioritise public health spending, the use of different data sources to estimate the healthcare costs of stroke and of diabetes will result in a bias towards one or other intervention and mean that an informed funding decision cannot be made. Despite this problem, published public health economic models commonly use cost estimates from a range of incomparable sources [7–9].

A second challenge is how to quantify the age and sex specific future economic consequences of individuals becoming unwell from diseases unrelated to those explicitly modelled following interventions that may prolong life [10,11]. Including such diseases in cost-effectiveness models may be important to understand the true consequences of a given intervention. The NICE guidelines manual recommends that all relevant costs and benefits should be included in public health economic analyses, although quantifying future costs and utility decrements resulting from unrelated diseases are not explicitly mentioned [2]. Others have suggested that their absence might lead to less “robust and defensible analyses”, and “suboptimal decisions” [11,12].

The aim of this paper is to describe a method for estimating comparable disease specific and unrelated future healthcare costs using routinely available data in England for use in health and public health economic modelling. We present both the method and the results of applying this method to 11 diseases included in an existing multistate life table model.

## Methods

A case study is used to illustrate the methodology: disease specific costs are estimated for the 11 diseases simulated by the multistate life table model, PRIMEtime (see supplementary data from Cobiac et al. [13]), and for diseases unrelated to those modelled by age and sex.

Disease specific costs are derived from 2013/14 NHS England programme budgeting data which reports expenditure by clinical commissioning groups (CCGs, responsible for commissioning local NHS health care services in England including mental health services, urgent and emergency care, elective hospital care, and community care), and accounts for around two thirds of the total NHS England budget [14]. Expenditure is collected using the same methodology for 56 disease categories and 15 care settings.

The majority of the remaining 2013/14 NHS England expenditure is accounted for by specialised services and primary care. Specialised services are commissioned nationally and are responsible for around 15% of the total NHS England budget; these are healthcare services that are either particularly expensive or have a small patient population [15]. Primary care is also paid for nationally (except primary care prescribing which is accounted for by programme budgeting data) and makes up approximately 12% of total NHS England spend.

### Allocating NHS expenditure to specific diseases

In 2013/14, programme budgeting data reported a spend of £63.4bn by CCGs, out of a total NHS England budget of £95.6bn [16]. Of the remainder, £13.4bn was allocated to specialised services and £11.3bn to primary care, with the rest spent on functions such as CCG running costs and public health functions [15].

**Fig 1:**
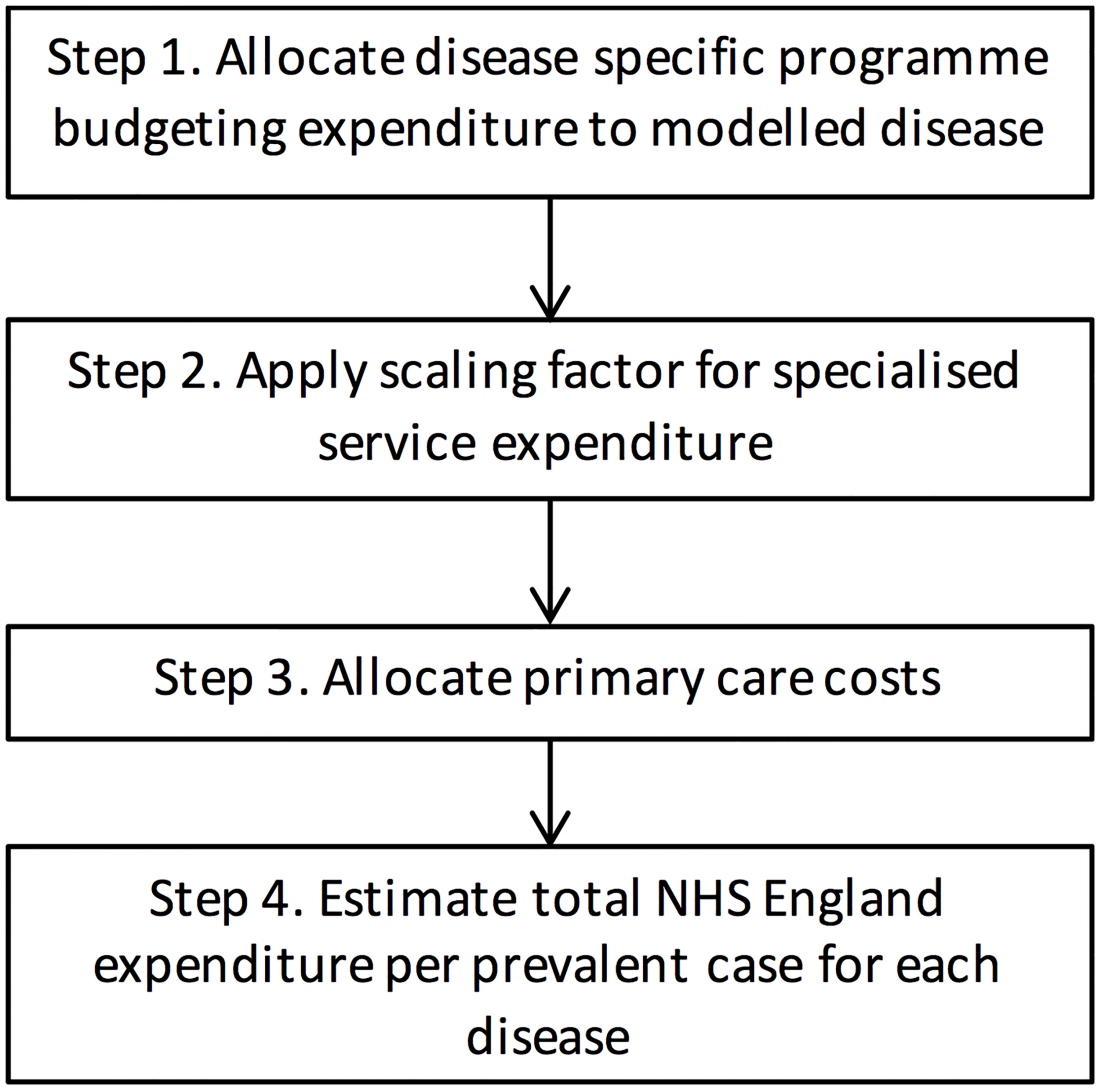
Flow chart describing the steps taken to assign disease specific expenditures.

Fig 1 shows the steps described in this manuscript to allocate NHS England expenditure to different diseases.

#### Step 1. Allocate disease specific programme budgeting expenditure

Table 1 shows the ICD-10 codes for the 11 modelled diseases in the case study, alongside the ICD-10 codes for the related programme budgeting category [17].

**Table 1:** The ICD-10 codes of modelled diseases alongside the ICD-10 codes of programme budgeting categories.

Modelled disease	Disease ICD-10 codes	Relevant programme budgeting category	Programme budgeting ICD-10 codes (ICD-10 codes that are the same as the modelled disease are in bold)
**Ischaemic heart disease**	I20-I25	10a Coronary Heart Disease	**I20-I25**, Z03.4, Z50.0, Z82.4, Z95.5
**Stroke**	I60-I69	10b Cerebrovascular disease	G46.0-G46.2, G46.8, **I60-I69**, Z82.3
**Type two diabetes**	E11, E14	04a Diabetes	E10, **E11**, E12, E13, **E14**, M14.2, Z13.1, Z83.3
**Breast cancer**	C50	02f Cancer, breast	**C50**, D05, Z12.3, Z80.3, Z85.3
**Colon cancer**	C18-C20	02c Cancer, lower GI	C17, **C18-C20**, C21, C26, Z12.1
**Lung cancer**	C34	02d Cancer, lung	C33, **C34**, C37-39, C45, Z80.1, Z80.2, Z85.1, Z85.2
**Stomach cancer**	C16	02b Cancer, upper GI	C15, **C16**, C22-C25, Z12.0
**Liver cancer**	C22	02b Cancer, upper GI	C15, C16, **C22**, C23, C24, C25, Z12.0
**Kidney cancer**	C64	02h Cancer, urological	C60-C63, **C64**, C65-C68, Z12.5
**Pancreatic cancer**	C25	02b Cancer, upper GI	C15, C16, C22, C23, C24, **C25**, Z12.0
**Liver disease**	K70, K74	13c Hepatobiliary	A06.4, A27.0, A95, B15-B19, B25.2, B25.2, B26.3, B58.1, B67.0, B67.5, I85, I86.4, I98.2, **K70**, K71, K72, K73, **K74**, K75, K76, K77, K80-K83, K85-K87, Q44, Q45, R16, R17, R82.2, R93.2, R94.5, Z22.5, Z52.6, Z94.4

IHD, ischaemic heart disease; GI, gastrointestinal

**Fig 2:**
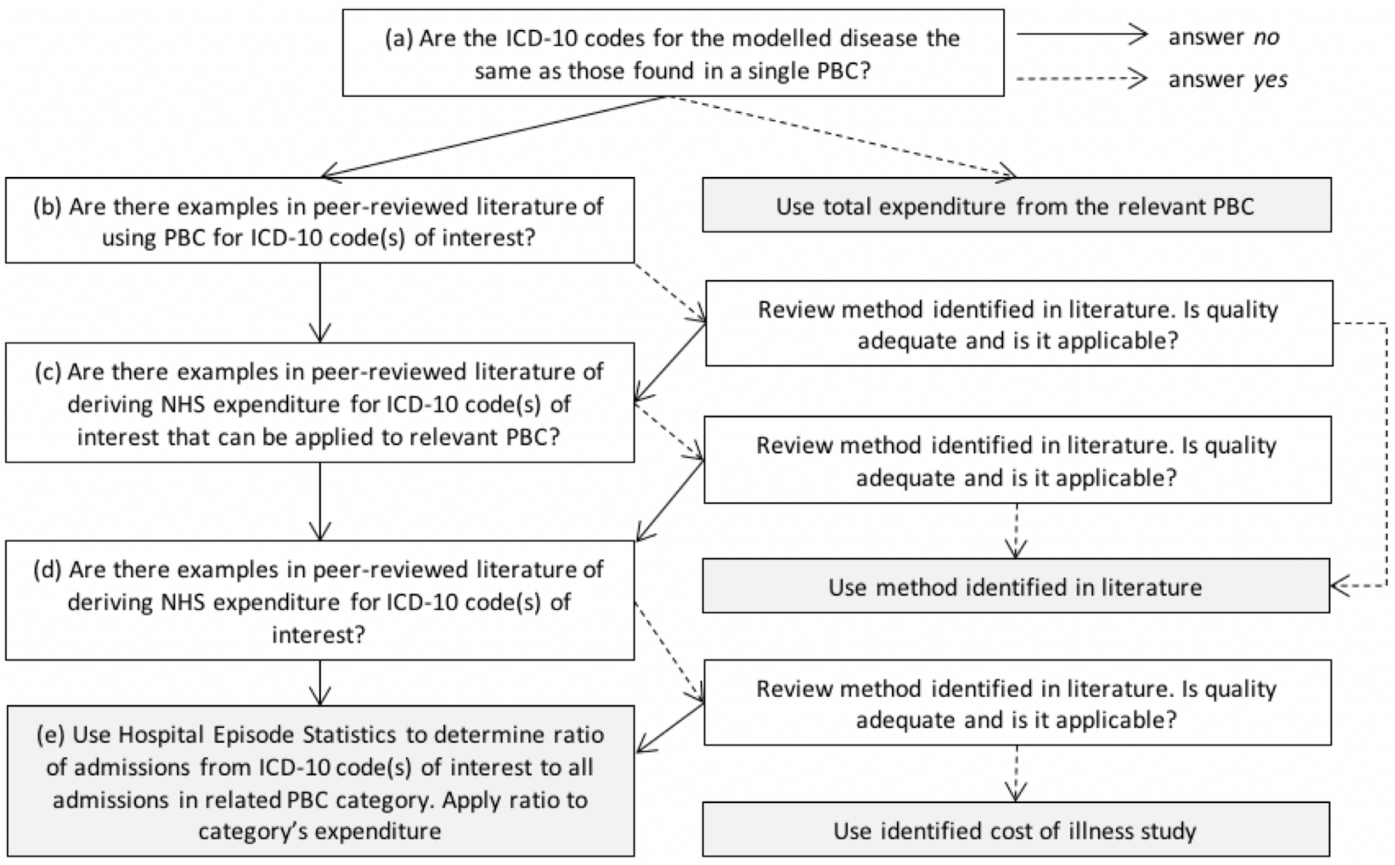
Flow chart for how to allocate expenditure associated with a programme budgeting category to ICD-10 codes for modelled diseases. PBC, programme budgeting category.

A review of the literature using “programme budgeting” in MEDLINE did not identify any peer-reviewed publications that have previously divided programme budgeting category expenditure into their component diseases. The UK Health Forum used 2012/13 programme budgeting data to quantify NHS England costs for some of their modelled diseases (but not all) based on incidence and prevalence ratios of diseases within each programme budgeting category [7,18]. Using their methodology as a baseline framework, the method shown in Fig 2 was developed to assign expenditure from programme budgeting categories to all individual diseases. Costs allocated to programme budgeting categories *21*, *healthy individuals; 22*, *social care needs*; and *23*, *other* are not included.

Part (a) in Fig 2 asks whether the ICD-10 codes for a modelled disease are the same as a programme budgeting category. If so, the total expenditure in that programme budgeting category is used. Parts (b) and (c) identify published literature that directly apportions expenditure for diseases within a programme budgeting category of interest by ICD-10 code thereby allowing the proportion of expenditure from the relevant modelled disease to be identified. In the absence of any papers being identified in parts (b) and (c), part (d) searches for papers containing an overall estimate of NHS expenditure for relevant ICD-10 codes which can be used in place of programme budgeting expenditure data. See Appendix A in S1 File for an illustrative search strategy.

Retrieved papers are appraised for whether they are relevant, applicable, and of adequate quality using quality criteria described in the Appendix A in S1 File. In the case of more than one relevant study being identified, the study with the highest quality score is used. Where more than one study has the same high quality score, the results of using each study are estimated and separately reported as a sensitivity analysis. We do not recommend combining data or using a range (with modelled distribution) due to the likely heterogeneity of methods used to identify costs.

Part (e) disaggregates expenditure within programme budgeting categories based on the ratio of admissions from each modelled disease ICD-10 codes to admissions from all ICD-10 codes in the relevant programme budgeting category using routinely available hospital episode statistics (HES) data [19]. Using stomach cancer as an example, this is the number of admissions from ICD-10 code C16 divided by total number of admissions from programme budgeting category *02b*, *Cancer*, *upper GI* ICD-10 codes C15, C16, C22-C25, and Z12.0. This is then multiplied by the programme budgeting expenditure for the entirety of category 02b.

#### Step 2. Specialised services expenditure

Specialised services expenditure data in 2013/14 are not available by individual disease [15]. Therefore 2012/13 programme budgeting data are used to estimate disease specific specialised services expenditure, when Primary Care Trusts (PCTs) were responsible for their commissioning, with costs reported under the 2012/13 care setting ‘Other Secondary Care’ [20]. Before 2013/14, local primary, secondary, and community health services in England were commissioned by PCTs. They were abolished in 2013 following the enactment of the 2012 Health and Social Care Act with CCGs subsequently taking responsibility for commissioning services.[21] This changed how the English NHS budget was organised with specialised services and primary care expenditure (except for primary care prescribing) subsequently being allocated nationally rather than locally. Therefore, specialised services expenditure was reported in programme budgeting data when PCTs were responsible for commissioning services (prior to 2013/14) and not after they were abolished.

To estimate specialised services expenditure for each disease, the ratio of 2012/13 programme budgeting expenditure on ‘Other Secondary Care’ to total 2012/13 programme budgeting expenditure for each relevant programme budgeting disease category is first calculated (not including expenditure on Prevention and Health Promotion, Other Secondary Care, and Primary Care as these care settings are not included in 2013/14 data). This ratio is then multiplied by the 2013/14 expenditure calculated in step one to estimate the specialised services expenditure by modelled disease.

The ratio used for all modelled cancer subtypes is calculated based on the whole of category *02*, *cancers and tumours* rather than the cancer subtype programme budgeting category because the majority of ‘Other Secondary Care’ expenditure for chemotherapy and radiotherapy is allocated to category *02x*, *cancers and tumours*, *other* rather than to each specific cancer subtype.[20]

For example, in 2012/13 programme budgeting data £1,407m was spent on ‘Other Secondary Care’ for category *02*, *cancers and tumours*. In the same year, £4,114m was spent on the cancers and tumours in settings that are the same as those reported in 2013/14 programme budgeting data (all settings except Primary Care, Prevention and Health Promotion, and Other Secondary Care). The ratio of ‘Other Secondary Care’ to total category costs for cancers and tumours in 2012/13 programme budgeting data was £1,407m divided by £4,114m (0.34). This was then used to calculate the specialist care costs for each cancer subtype included in PRIMEtime by multiplying 0.34 with the total 2013/14 disease-specific programme budgeting spend calculated in step 1. For example, the spend on colon cancer calculated in step 1 was £248m, so 2013/14 specialised services expenditure on colon cancer was estimated to be £85m (£248m multiplied by 0.34).

#### Step 3. Allocating primary care costs

Primary care expenditure (except for money spent on prescribing) is not included in programme budgeting data. To calculate the primary care expenditure (not including prescribing costs) for each disease, primary care prescribing expenditure data reported in programme budgeting is used. For each programme budgeting category, total primary care prescribing expenditure is multiplied by the proportion of expenditure within the category that is related to the modelled disease calculated in step one (e.g. the proportion of programme budgeting category *02b*, *Cancer*, *upper GI* that is allocated to stomach cancer). The result is then divided by total primary care prescribing expenditure across all diseases and multiplied by 2013/14 primary care total expenditure. This figure is added to total costs from steps one and two to give the final disease specific expenditure for NHS England in 2013/14. This method assumes that primary care expenditure on a given disease is proportional to the amount spent on primary care prescribing.

For example, in the case study £764m was spent on primary care prescribing for programme budgeting category 04a, diabetes in 2013/14. This was multiplied by the proportion of category 04a that was related to type two diabetes (0.90) and divided by total primary care prescribing expenditure in 2013/14 (£8,035bn). The result (0.085) is the proportion of total primary care prescribing expenditure spent on type two diabetes and was multiplied by primary care total expenditure (£11.3bn) to estimate a 2013/14 primary care spend on type two diabetes of £960m.

#### Step 4. Estimating expenditure per prevalent case

For each modelled disease, total costs estimated in the previous steps are divided by the 2014 disease prevalence to estimate the 2013/14 cost per prevalent case in England. In the case study, 2014 disease prevalence data were as estimated by PRIMEtime [13].

### Estimating NHS England expenditure per person by age and sex for unrelated diseases

Future expenditure per person by age and sex for diseases unrelated to those modelled are estimated using NHS England cost curves [22]. Cost curves are a by-product of NHS England’s CCG resource allocation formulae and describe the relative health expenditure by age group and by sex for four care categories—general and acute care, mental health, prescribing, and primary care. Specialised services and maternity expenditure are not used in the derivation of cost curves because these are not commissioned by CCGs. However, they are included in method described here to estimate the total expenditure for diseases unrelated to those modelled.

For acute care, mental health, and prescribing, ratios of expenditure compared to the most expensive age and sex group are directly calculated from the published curves. For primary care, NHS England cost-curves report the average time in minutes per year spent with a GP compared to a 0–4 year old male (set to 0 minutes). The average time spent in minutes is used as a marker of intensity of primary care use and therefore costs. To calculate the ratios of time spent with a GP for each age and sex group compared to the age and sex group spending the most amount of time, the baseline length of time spent in primary care for a male aged 0–5 years is taken from the NHS England primary medical care allocation formula (34.7 minutes). The average time in minutes spent per year with a GP is calculated for each age and sex group so that the ratio can be estimated [23]. Calculated ratios from each of the four NHS England cost curves are shown in Table B in S1 File.

To estimate age and sex specific NHS England expenditure on unrelated diseases, NHS England expenditure on ICD-10 codes from unrelated disease is calculated by subtracting the total expenditure on modelled diseases estimated in step four from NHS England’s budget for clinical services (CCGs, specialised services, and primary care). The remaining NHS England expenditure, except for maternity services, is divided into the four care categories for which cost-curves are available. Non-disease specific NHS England costs including running costs, surplus, and PHE costs, as well as costs from programme budgeting categories 21 (healthy individuals), 22 (social care needs), and 23 (other) are not included.

To allocate programme budgeting expenditure on unrelated diseases to the four cost curves, programme budgeting categories *5*, *mental health disorders* and *6*, *problems of learning disability* are allocated to mental health, and all prescribing costs (programme budgeting care settings, *primary care prescribing* and *unbundled/high cost*: *drugs and devices*) are allocated to prescribing. Remaining programme budgeting expenditure, except for category *18*, *maternity and reproductive health*, is allocated to general and acute care. All specialised services expenditure is allocated to general and acute care except for the proportion spent on mental health, estimated using the method described in step two above. The £9.8bn spent on primary care is allocated to primary care except for a proportion allocated to mental health, calculated using the method in step three above. Finally, programme budgeting expenditure on category *18*, *maternity and reproductive health* is allocated based on the proportion of total 2014 live births by mothers’ age in England and Wales [24]. The ratios used to allocate maternity costs by age and sex can be found in Table B in S1 File.

Total annual unrelated NHS England expenditure by age and sex is the sum of the costs allocated according to each cost curve described above plus maternity services for each age and sex group.

## Results

### Disease specific NHS England expenditure

Total NHS England expenditure for the case study’s modelled diseases is shown in Table 2, with the largest categories of expenditure being type two diabetes (£2,057m) and ischaemic heart disease (IHD) (£1,481m). Using PRIMEtime disease prevalence rates, the annual excess cost to NHS England per prevalent case varied between £3,074 for pancreatic cancer and £314 for liver disease (Table 2). These costs are the average costs across all individuals with the disease, irrespective of time since diagnosis.

**Table 2:** Total 2013/14 expenditure by modelled disease (£000s except cost per prevalent case).

Modelled disease	Programme budgeting category	Programme budgeting expenditure	Specialised services expenditure	Primary care expenditure	Total NHS England disease costs	Annual cost per prevalent case (£)
		Step 1	Step 2	Step 3		
**Ischaemic heart disease**	10a Coronary Heart Disease	953,743	41,818	485,056	1,480,617	1,905
**Stroke**.	10b Cerebrovascular disease	689,876	55,443	29,475	774,794	843
**Type two diabetes**	04a Diabetes	1,071,537	25,577	959,716	2,056,831	444
**Breast cancer**	02f Cancer, breast	472,192*	N/A	N/A	472,192	573
**Colon cancer**	02c Cancer, lower GI	248,315	84,919	20	333,253	810
**Lung cancer**	02d Cancer, lung	98,250	33,599	0†	131,849	904
**Stomach cancer**	02b Cancer, upper GI	32,794	11,215	0†	44,008	535
**Liver cancer**	02b Cancer, upper GI	16,990	5,810	0†	22,801	1,532
**Kidney cancer**	02h Cancer, urological	25,145	8,599	7,833	41,577	618
**Pancreatic cancer**	02b Cancer, upper GI	42,133	14,409	0†	56,542	3,074
**Liver disease**	13c Hepatobiliary	59,702	4,543	2,963	67,209	314

*Breast cancer costs not estimated from programme budgeting expenditure (see text). GI, gastrointestinal; N/A, not applicable;

^†^primary care costs estimated to be negligible

In order to allocate programme budgeting expenditure to modelled diseases (Fig 2), part (a) was used for IHD and stroke, where ICD-10 codes were the same as programme budgeting categories *10a*, *coronary heart disease* and *10b*, *cerebrovascular disease* respectively. There were some additional ICD-10 codes included in programme budgeting categories 10a and 10b not included in PRIMEtime (Table 1), however these were responsible for just 0.01% and 0.02% of all HES admissions within categories 10a and 10b respectively in 2013/14 and were therefore disregarded. There were no relevant papers identified for any disease of interest following part (b), and at part (c) a paper by Hex and colleagues was used for the ratio of direct NHS expenditure on type one diabetes to type two diabetes [25]. This ratio of 0.10 was applied to the 2013/14 expenditure in programme budgeting category 04a to estimate type two diabetes costs. No other relevant publications of adequate quality were identified for any other diseases of interest in either part (b) or (c).

In part (d), a paper by Luengo-Fernandez et al. was used to estimate breast cancer costs, including primary care, emergency care, outpatient care, hospital inpatient care, and drugs (ICD-10 codes in the paper for lung, colorectal, and prostate cancer are not the same as those modelled) [26]. Total 2009 UK healthcare costs from Luengo-Fernandez were adjusted to 2013/14 English healthcare costs by scaling results to the English population [27], converting from Euros to pounds sterling using 2009 exchange rates [28], and inflating to 2013/14 costs using the hospital and community health services (HCHS) index giving a total cost of £472m [29].

For the remaining modelled diseases, expenditure within the related programme budgeting categories were derived using part (e), based on 2013/14 HES admissions data [19].

### NHS England expenditure per person from unrelated diseases

**Fig 3:**
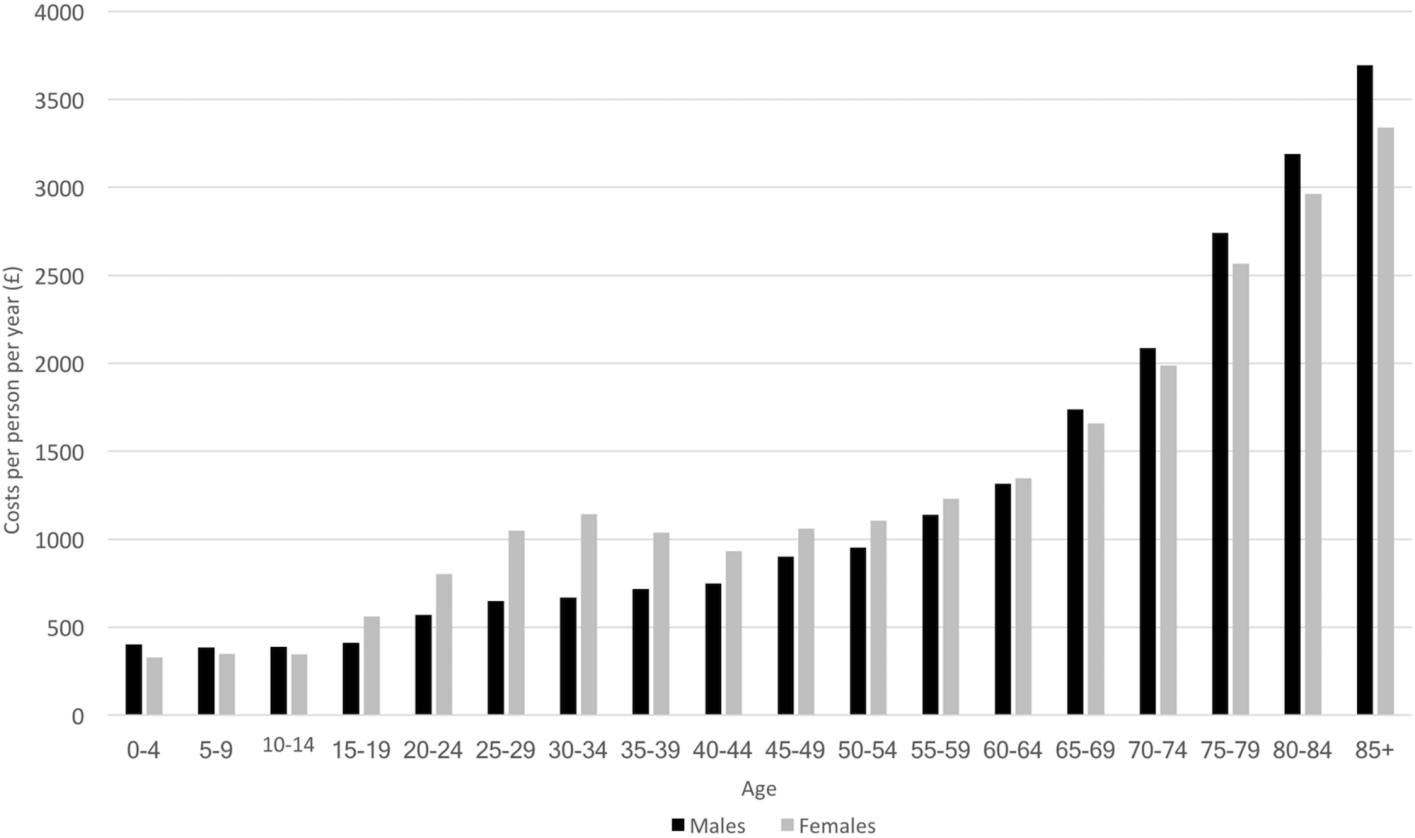
NHS England annual per person expenditure on unrelated diseases to those modelled by age and sex.

Annual expenditure on unrelated diseases to those modelled is shown in Table 3 by care category and category of expenditure. Costs increased with age, except for a secondary peak at 30–34 years for women reflecting maternity resource use (Fig 3). Expenditure by age and sex is in Table C in S1 File.

**Table 3:** NHS England expenditure on unrelated diseases by care category and by category of expenditure (£000s).

	General and acute	Mental health	Prescribing	Primary care	Maternity	Total
Programme budgeting	27,583,622	8,699,759	7,647,576	0	2,660,078	46,591,034
Specialised services	7,000,830	5,987,496	0	0	0	12,988,326
Primary care	0	931,601	0	8,818,666	0	9,750,267
Total	34,584,451	15,618,857	7,647,576	8,818,666	2,660,078	69,329,627

## Discussion

We describe a method for estimating comparable English healthcare costs across multiple modelled diseases, and future costs arising from unrelated diseases, using routinely available data. The benefit of this approach is that limitations are shared by each disease included in the model and should not bias outcomes in one particular direction.

### Assumptions and limitations

The accuracy of programme budgeting data is directly related to how CCGs report their expenditure. Expenditure varies by CCG due to local population size, need, and availability of services, and although programme budgeting data reliability has been challenged [30], systematic reporting errors are thought to be unlikely [31].

Category 23 (other) was responsible for 20% of all 2013/14 programme budgeting expenditure but not included when estimating disease specific expenditure meaning overall expenditure by disease may be underestimated. The majority of these costs are in the subcategories *23d*, *condition not known*, *23f*, *condition data not recorded/reported*, and *23x*, *miscellaneous other*. Although some diseases may be more likely to have associated costs coded under category 23 than others, it is not possible to estimate which diseases are more affected.

For the majority of modelled diseases, HES admissions data were used to estimate the relative disease burden within a given programme budgeting category. This assumes that disease burden is proportional to secondary care admissions and that the cost per admission is the same for each disease within a programme budgeting category. This may underestimate total NHS England expenditure on diseases managed more in an outpatient setting compared to other diseases in the same category, and vice versa. An example is diabetes, where 44% of HES admissions within the programme budgeting category *04a*, *diabetes* report type two diabetes as the cause of admission with type one diabetes accounting for most other admissions [19]. However, Hex et al. (used in this case study) estimate that type two diabetes accounts for 90% of total diabetes costs [25].

The allocation of specialised services expenditure to modelled diseases assumes that the ratio of expenditure on ‘Other secondary care’ to the total spend for each disease by PCTs in 2012/13 is equivalent to the relative spend by each disease category on specialised services in 2013/14. This method was used because it is currently not possible to obtain data on specialised services expenditure for programme budgeting categories and there is no precedent in the literature. This method might over or underestimate true expenditure on different diseases. Personal communication with NHS England suggests that future data releases may include expenditure by specialised service.

Primary care costs were estimated assuming that they are proportional to primary care prescribing expenditure. Repeating the analysis using HES admissions meant that primary care costs from diabetes were lower compared to using primary care prescribing data, and cancer costs were higher (results not shown). It is likely that using HES admissions underestimate primary care costs for diseases managed primarily in the community such as diabetes, and overestimate costs from diseases managed predominantly by specialist centres such as cancer. This is in keeping with estimates that suggest just 2.5% of breast cancer costs occur outside of secondary care [32].

Future NHS England costs from unrelated diseases assume that cost-curves represent how specialised services expenditure varies by age and sex [22]. It is likely that too little was allocated to the prescribing cost curve as data were not available on how much of each specialised service was spent on prescribing. This may under-estimate unrelated disease costs among older individuals as the prescribing cost-curve more heavily weights costs towards those aged between 60 and 80 years than the general and acute services cost-curve.

Finally, NHS England cost-curves are derived using all diseases however they are used to apportion NHS costs only to unrelated diseases, thereby not including the modelled diseases which in PRIMEtime are all more common among older age groups. This may under-estimate unrelated disease costs for younger individuals and over-estimate them for older individuals.

### Comparisons with other studies

The only disease for which a cost of illness study from the peer-reviewed literature of sufficient quality was identified was breast cancer [26]. If costs were instead calculated using HES admissions data (as with other cancers quantified in this study, using part (e) of step 1 in Fig 2), total costs would be £469,771,000, very similar to the £472,192,000 estimated by Luengo-Fernandez et al. using bottom-up methods. We would recommend that if appropriate cost of illness studies are identified using the methods described in this paper, the costs of the disease using part (e) should also be estimated for comparison. Sensitivity analyses could then be used to quantify the impact of any significant differences in costs on modelled cost-effectiveness results.

Annual NHS costs per prevalent case have been used by other health economic studies modelling multiple diseases, for example, Trueman and Anokye [8], Trueman et al. [33], and Frew et al. [9]. Each study used different sources for cost estimates ranging from £817 to £1,934 per year for diabetes, £114 to £2,047 for CHD, and £415 to £2,591 for stroke (all costs converted to 2014 healthcare costs using the HCHS index [29]). Cost estimates per case for CVDs are in line with those estimated in this paper, however diabetes costs are considerably higher. The annual cost of £1,934 per case used by Trueman and Anokye is based on a 1994/5 estimate of the excess cost of diabetic patients admitted in South Glamorgan Health Authority [8,34]. Although the total cost may represent the additional burden on acute care among diabetic patients compared with non-diabetic patients, it calculates costs associated with all diabetic patients including those being admitted for co-morbid conditions such as CVD. These co-morbid costs are captured using the methods in this paper through either modelled diseases (such as IHD and stroke) or unrelated disease costs, thus avoiding double counting.

Frew et al. reported annual costs per prevalent case for colorectal cancer to be £10,814 (based on a report estimating the total annual cost of colorectal cancer in England at £1,326m, 2014 prices [35]) [9], and Trueman et al. used an annual colorectal cancer cost of £9,154 per case. These are both significantly higher than results in Table 2 and were estimated using bottom-up methods. The annual total used by Frew et al. compares to just £333m reported in Table 3 and £487m for colorectal and anal cancer estimated by Luengo-Fernandez et al. (2014 prices) [26]. The differences may be due to other studies not including low long-term costs of those who have historically had a disease and remain a prevalent case but die of an unrelated cause. And as with diabetes cost estimates, costs attributed to the cancer diagnosis in these reports may be due to co-morbid conditions which are quantified though modelled and unrelated diseases.

Finally, in the absence of any UK studies estimating unrelated healthcare costs by age and sex, results are compared with Blakely et al. who estimated healthcare expenditure for diseases unrelated to tobacco consumption for the New Zealand population by age and sex using bottom-up unit patient care costs [36]. The magnitude of expenditure pattern by age is similar to that shown in Fig 3, except for those aged under four years. Blakely et al. estimated significantly higher costs attributable to unrelated diseases in the first four years of life, and the methods in this paper may underestimate these costs.

### Conclusions

In summary, we describe a novel approach to estimating NHS England costs for multiple diseases and unrelated future healthcare costs using routinely available data. The key strength is that we use a consistent approach to estimating costs across multiple diseases meaning that the cost implications of different interventions affecting these diseases can be directly compared. This approach can be applied to other health and public health economic models that estimate the economic consequences of an intervention affecting multiple diseases in England.

## Supporting information

S1 File Supplementary data file. (DOCX)
