# Supplementary material for: Estimating comparable English healthcare costs for multiple diseases and unrelated future costs for use in health and public health economic modelling
Source: PLoS One. 2018 May 24;13(5):e0197257. doi: 10.1371/journal.pone.0197257 (PMC5967835; doi:10.1371/journal.pone.0197257)
Supplement: S1 File — (DOCX) [file pone.0197257.s001.docx]

**Estimating comparable English healthcare costs for multiple diseases and unrelated future costs for use in health and public health economic modelling**

**S1 File. Supplementary Data.**

**Appendix A. Illustrative search strategy used for parts (b), (c), and (d) of figure 2**

For the case study diseases, the following search strategy was used for parts (b), (c), and (d) for each modelled disease (using liver disease as an example):

Database: Ovid MEDLINE(R) In-Process & Other Non-Indexed Citations and Ovid MEDLINE(R) 1946 to Present

Search terms:

1. exp Liver Diseases/
2. exp Great Britain/
3. (“National Health Service” OR NHS).mp.
4. exp “Costs and Cost Analysis”/
5. 1 and (2 or 3) and 4

***Quality checklist for assessing studies identified in figure 2.***

Papers identified following literature searches in parts (b), (c), and (d) of figure 1 were assessed for relevance, applicability, and quality using the study screening questions below and the scoring system in table S1.

The quality checklist is based on a similar example for costing studies developed by Luengo-Fernandez [1], as well as the checklist for assessing economic evaluations in chapter three of Drummond et al.’s Methods for the Economic Evaluation of Health Care Programmes, The Consolidated Health Economic Evaluation Reporting Standards (CHEERS) statement, ISPOR-SMDM Good Research Practices for Measuring Drug Costs, and the Critical Appraisal Skills Programme (CASP) appraisal tool for economic evaluations [2–5].

*Study screening questions*

If answer no to one of the questions below, then disregard study.

1. Does the study include estimation of UK or English healthcare costs covering ICD-10 codes of interest?
2. Is the year of analysis 2001 or later?

**Table A.** Study quality checklist questions

| Item no. | Criteria | Yes/Partially/No  (score 2/1/0) |
| --- | --- | --- |
| 1 | Does the study state economic perspective? |  |
| 2 | If estimated, are indirect and direct costs reported separately? |  |
| 3 | Are methods described for cost and valuation estimates? |  |
| 4 | Are all relevant healthcare costs included, or if not, is it clear as to which are and which are not? |  |
| 5 | Are currency, price data, conversion rates, and medical care inflation calculations reported and appropriate? |  |
| 6 | Is the time horizon stated, and are annual costs available or can they be derived? |  |
| 7 | Are discount rates stated where appropriate? |  |
| 8 | Is the relevant uncertainty around costs reported? |  |
| 9 | What is the year of analysis (score 0 if 2001-2006, 1 if 2006-2011, 2 if >2011)? |  |

The quality checklist is based on a similar example for costing studies developed by Luengo-Fernandez [1], as well as the checklist for assessing economic evaluations in chapter three of Drummond et al.’s Methods for the Economic Evaluation of Health Care Programmes, The Consolidated Health Economic Evaluation Reporting Standards (CHEERS) statement, ISPOR-SMDM Good Research Practices for Measuring Drug Costs, and the Critical Appraisal Skills Programme (CASP) appraisal tool for economic evaluations [2–5].

**Appendix B. Additional data.**

**Table B.** Ratios used for estimating age and sex specific NHS England expenditure on diseases unrelated to those modelled for each category of expenditure

| **Age group** | **General and acute*** | **Mental health*** | **Prescribing*** | **Primary care*** | **Maternity†** |
| --- | --- | --- | --- | --- | --- |
| **M0-4** | 0.110 | 0.001 | 0.088 | 0.219 | 0.000 |
| **M5-9** | 0.078 | 0.253 | 0.080 | 0.078 | 0.000 |
| **M10-14** | 0.079 | 0.253 | 0.080 | 0.078 | 0.000 |
| **M15-19** | 0.080 | 0.244 | 0.106 | 0.111 | 0.000 |
| **M20-24** | 0.081 | 0.554 | 0.106 | 0.111 | 0.000 |
| **M25-29** | 0.079 | 0.714 | 0.115 | 0.111 | 0.000 |
| **M30-34:** | 0.083 | 0.730 | 0.115 | 0.111 | 0.000 |
| **M35-39** | 0.095 | 0.737 | 0.159 | 0.111 | 0.000 |
| **M40-44** | 0.120 | 0.682 | 0.159 | 0.111 | 0.000 |
| **M45-49** | 0.152 | 0.606 | 0.274 | 0.262 | 0.000 |
| **M50-54** | 0.190 | 0.525 | 0.274 | 0.262 | 0.000 |
| **M55-59** | 0.251 | 0.457 | 0.469 | 0.262 | 0.000 |
| **M60-64** | 0.330 | 0.425 | 0.469 | 0.262 | 0.000 |
| **M65-69** | 0.409 | 0.452 | 0.770 | 0.479 | 0.000 |
| **M70-74** | 0.536 | 0.532 | 0.770 | 0.479 | 0.000 |
| **M75-79** | 0.682 | 0.729 | 1.000 | 0.728 | 0.000 |
| **M80-84** | 0.830 | 0.904 | 1.000 | 0.728 | 0.000 |
| **M85+** | 1.000 | 0.877 | 1.000 | 0.957 | 0.000 |
| **F0-4** | 0.084 | 0.001 | 0.080 | 0.199 | 0.000 |
| **F5-9** | 0.063 | 0.253 | 0.062 | 0.087 | 0.000 |
| **F10-14** | 0.072 | 0.208 | 0.062 | 0.087 | 0.000 |
| **F15-19** | 0.097 | 0.208 | 0.124 | 0.277 | 0.120 |
| **F20-24** | 0.107 | 0.327 | 0.124 | 0.277 | 0.522 |
| **F25-29** | 0.120 | 0.430 | 0.159 | 0.277 | 0.913 |
| **F30-34** | 0.134 | 0.478 | 0.159 | 0.277 | 1.000 |
| **F35-39** | 0.147 | 0.511 | 0.230 | 0.277 | 0.534 |
| **F40-44** | 0.168 | 0.511 | 0.230 | 0.277 | 0.125 |
| **F45-49** | 0.200 | 0.531 | 0.327 | 0.382 | 0.009 |
| **F50-54** | 0.232 | 0.477 | 0.327 | 0.382 | 0.000 |
| **F55-59** | 0.264 | 0.456 | 0.478 | 0.382 | 0.000 |
| **F60-64** | 0.317 | 0.429 | 0.478 | 0.382 | 0.000 |
| **F65-69** | 0.377 | 0.487 | 0.673 | 0.523 | 0.000 |
| **F70-74** | 0.483 | 0.630 | 0.673 | 0.523 | 0.000 |
| **F75-79** | 0.594 | 0.841 | 0.876 | 0.784 | 0.000 |
| **F80-84** | 0.725 | 1.000 | 0.876 | 0.784 | 0.000 |
| **F85+** | 0.854 | 0.926 | 0.876 | 1.000 | 0.000 |

*Ratios derived from NHS England cost curves[6]

**†**Ratios based on the proportion of total 2014 live births by mothers’ age in England and Wales[7]

**Table C.** Annual expenditure on diseases unrelated to those modelled by age and sex and by category of expenditure (£)

| **Age group** | **General and acute*** | **Mental health*** | **Prescribing*** | **Primary care*** | **Maternity†** | **Total** |
| --- | --- | --- | --- | --- | --- | --- |
| **M0-4** | 267 | 0 | 34 | 101 | 0 | 403 |
| **M5-9** | 189 | 129 | 30 | 36 | 0 | 384 |
| **M10-14** | 192 | 129 | 30 | 36 | 0 | 387 |
| **M15-19** | 194 | 124 | 40 | 51 | 0 | 410 |
| **M20-24** | 196 | 282 | 40 | 51 | 0 | 569 |
| **M25-29** | 191 | 363 | 44 | 51 | 0 | 649 |
| **M30-34:** | 202 | 371 | 44 | 51 | 0 | 668 |
| **M35-39** | 230 | 375 | 60 | 51 | 0 | 716 |
| **M40-44** | 290 | 346 | 60 | 51 | 0 | 748 |
| **M45-49** | 369 | 308 | 104 | 121 | 0 | 902 |
| **M50-54** | 461 | 267 | 104 | 121 | 0 | 952 |
| **M55-59** | 608 | 232 | 178 | 121 | 0 | 1139 |
| **M60-64** | 801 | 216 | 178 | 121 | 0 | 1316 |
| **M65-69** | 993 | 230 | 292 | 222 | 0 | 1737 |
| **M70-74** | 1301 | 270 | 292 | 222 | 0 | 2085 |
| **M75-79** | 1655 | 371 | 379 | 337 | 0 | 2742 |
| **M80-84** | 2014 | 459 | 379 | 337 | 0 | 3189 |
| **M85+** | 2427 | 446 | 379 | 443 | 0 | 3694 |
| **F0-4** | 204 | 0 | 30 | 92 | 0 | 327 |
| **F5-9** | 154 | 129 | 24 | 40 | 0 | 346 |
| **F10-14** | 175 | 106 | 24 | 40 | 0 | 344 |
| **F15-19** | 234 | 106 | 47 | 128 | 46 | 562 |
| **F20-24** | 260 | 166 | 47 | 128 | 201 | 803 |
| **F25-29** | 290 | 219 | 60 | 128 | 352 | 1049 |
| **F30-34** | 325 | 243 | 60 | 128 | 386 | 1142 |
| **F35-39** | 356 | 260 | 87 | 128 | 206 | 1037 |
| **F40-44** | 408 | 260 | 87 | 128 | 48 | 931 |
| **F45-49** | 485 | 270 | 124 | 177 | 4 | 1059 |
| **F50-54** | 563 | 243 | 124 | 177 | 0 | 1106 |
| **F55-59** | 640 | 232 | 181 | 177 | 0 | 1230 |
| **F60-64** | 769 | 218 | 181 | 177 | 0 | 1345 |
| **F65-69** | 915 | 247 | 255 | 242 | 0 | 1659 |
| **F70-74** | 1171 | 320 | 255 | 242 | 0 | 1989 |
| **F75-79** | 1443 | 427 | 332 | 363 | 0 | 2565 |
| **F80-84** | 1759 | 508 | 332 | 363 | 0 | 2962 |
| **F85+** | 2074 | 471 | 332 | 462 | 0 | 3339 |

**References**

1. Luengo-Fernandez R. Resource costs, health outcomes and cost-effectivenes in stroke care: evidence from the Oxford Vascular Study [Internet]. University of Oxford. 2009. Available: https://ora.ox.ac.uk/objects/uuid:a5012d51-6794-48a7-bb78-4e5166e8cfdf

2. Husereau D, Drummond M, Petrou S, Carswell C, Moher D, Greenberg D, et al. Consolidated Health Economic Evaluation Reporting Standards (CHEERS) statement. BMJ. 2013;346: f1049. doi:10.1136/bmj.f1049

3. Hay JW, Smeeding J, Carroll N V, Drummond M, Garrison LP, Mansley EC, et al. Good research practices for measuring drug costs in cost effectiveness analyses: issues and recommendations: the ISPOR Drug Cost Task Force report-Part I. Value Health. 2010;13: 3–7. doi:10.1111/j.1524-4733.2009.00663.x

4. Critical Appraisal Skills Programme. CASP Economic Evaluation Checklist [Internet]. Oxford; 2013. Available: http://media.wix.com/ugd/dded87_3b2bd5743feb4b1aaac6ebdd68771d3f.pdf

5. Drummond MF, Sculpher MJ, Claxton K, Stoddart GL, Torrance GW. Methods for the Economic Evaluation of Health Care Programmes. 4th ed. Oxford: Oxford University Press; 2015.

6. NHS England. Technical Guide to Allocation Formulae and Pace of Change [Internet]. Leeds; 2016. Available: https://www.england.nhs.uk/wp-content/uploads/2016/04/1-allctins-16-17-tech-guid-formulae.pdf

7. Office for National Statistics. Births by Parents’ Characteristics, England and Wales: 2014 [Internet]. London; 2015. Available: https://www.ons.gov.uk/file?uri=/peoplepopulationandcommunity/birthsdeathsandmarriages/livebirths/datasets/birthsbyparentscharacteristics/2014/3birthsbyparentscharacteristics2014final.xls
